# Impact of nicotine and maternal BMI on fetal birth weight

**DOI:** 10.1186/s12884-021-03593-z

**Published:** 2021-02-12

**Authors:** Veronika Günther, Ibrahim Alkatout, Christoph Vollmer, Nicolai Maass, Alexander Strauss, Manfred Voigt

**Affiliations:** 1 Department of Obstetrics and Gynecology, University Hospitals Schleswig-Holstein, Campus Kiel, Arnold-Heller-Str. 3 (Building C), 24105 Kiel, Germany; 2 Christian-Albrechts-University zu Kiel, 24118 Kiel, Germany; 3 Department of Gynecology, University Hospital Freiburg, Hugstetter Str. 55, 79106 Freiburg, Germany; 4 Center for Medicine and Society, Albert-Ludwigs-University Freiburg, Friedrichstr. 39, 79098 Freiburg, Germany

**Keywords:** Pregnancy, Nicotine abuse, Body mass index, Birth weight

## Abstract

**Background:**

According to the World Health Organization, smoking is the most important risk factor for adverse pregnancy outcomes in industrialized nations. As the individual factors (body mass index – BMI (kg/m^2^) – and cigarette consumption) have been extensively investigated in pregnancy, we aimed to establish how maternal BMI and nicotine interact with regard to perinatal outcomes and birth weight.

**Methods:**

Data from 110.047 singleton pregnancies, achieved from the German Perinatal Survey in Schleswig-Holstein and registered between 2010 and 2017 were analyzed in August 2018 concerning maternal BMI and smoking. The BMI was taken from the maternity log. Information concerning the smoking status were self-reported and further subdivided into the following four categories: a) non-smokers; b) 1–7 cigarettes/day; c) 8–14 cigarettes/ day; and d) ≥ 15 cigarettes/ day.

Furthermore, we classified women by their BMI into underweight, normal weight, overweight and obese. Comparisons between non-smokers and the respective smoking group, and their relationship with maternal BMI were performed by the t-test (birth weight). A *P*-value ≤0.05 was considered to indicate statistical significance.

**Results:**

A number of 97.092 women (88.2%) were non-smokers and 12.955 (11.8%) were smokers. Furthermore 10.3% of women of normal weight smoked during pregnancy, but both high and low BMI were associated with a high prevalence of smoking. The proportion of smokers was highest (18.1%) among underweight women (BMI ≤ 18.5 kg/m^2^). A large number of smokers (15.5%) were registered in the obesity group (BMI ≥ 30 kg/m^2^).

Mean birth weight (≥ 37 + 0 gestational age) increased with increasing maternal BMI, and was reduced by smoking for every BMI category. The differences between smokers and non-smokers were always highly significant (*p* < 0.001). Mean birth weight varied between 2995 g in underweight frequent smokers and 3607 g in obese non-smokers.

**Conclusion:**

Both maternal BMI and smoking during pregnancy influences the birth weight and therefore pregnancy outcome. Smoking during pregnancy was significantly associated with low birth weight. Pregnant women should be advised to cease or at least reduce smoking in order to improve the birth weight of the newborn and to minimize child morbidities.

## Background

According to the World Health Organization, smoking is the most important risk factor for adverse pregnancy outcomes in industrialized nations [1, 2]. The association between maternal smoking and retarded fetal growth was first described in 1957, and is now a well-known fact [3]. Although smoking is associated with many health risks for mother and child, several thousands of pregnant women are known to smoke. A German Perinatal Quality Survey has shown that the percentage of pregnant smoking women in Germany is 10.9% [4]. In the European Union, it has been estimated that 10–27% of the pregnant women continue smoking during pregnancy [5] In comparison, in the USA the average for women who smoke during pregnancy is 7.1% [6]. Low birth weight, small for gestational age (SGA) status, preterm birth, and a low APGAR score are just a few of the harmful effects of nicotine on pregnancy and the newborn [7, 8]. The effects of nicotine are seen in every trimester of pregnancy; these range from increased spontaneous abortions in the first trimester to increased preterm delivery rates and a low birth weight in the final trimester [9]. A recent study from Rogers [10] showed that smoking causes an epigenetic phenomena because of altered DNA methylation. These epigenetic alterations are extensive and postnatally durable. A causal link between altered DNA methylation and the phenotypic changes observed in offspring remains to be firmly established, yet the association is strong, and mediation analyses suggest a causal link [10].

Many chemicals contained in cigarettes gain access to the fetal compartment via the placenta. In fact, fetal concentrations of these substances are generally 15% higher than maternal levels [11]. Nicotine narrows the blood vessels and also reduces blood flow to the placenta [11]. Carbon monoxide displaces oxygen from red blood cells, which leads to a chronic oxygen deficiency for the fetus [11]. As a result of poor circulation in the placenta, the unborn child receives fewer nutrients, resulting in a lower birth weight [11]. Cadmium is a particularly toxic component of tobacco smoke. The concentration of this heavy metal increases significantly with every cigarette smoked, both in maternal blood and umbilical cord blood [12]. The number of cigarettes consumed per day influences the child’s birth weight and increases the risk of preterm birth [13]. As the frequency of maternal smoking increases, the infant’s birth weight decreases. Low birth weight and preterm birth are associated with several complications, such as respiratory distress, feeding intolerance, and problems in neurodevelopment [14]. Cessation of smoking can at least partly reverse the negative effects on fetal growth. Apart from smoking, maternal BMI also influences the infant’s birth weight [15]. A mother being underweight or obese are both associated with adverse perinatal outcomes. Obese women have a higher rate of pregnancy-related complications, such as hypertension, gestational diabetes or preeclampsia, and are also subject to more fetal risks, such as macrosomia, structural fetal abnormalities or a lower APGAR score [16]. On the other hand, a low maternal BMI is associated with higher rates of preterm delivery and a higher risk of low birth weight [17]. While the individual factors involved in pregnancy (BMI with the extremes of overweight and underweight and cigarette smoking) are well investigated, we aimed to determine how maternal BMI and nicotine interact with regard to perinatal outcomes in terms of birth weight.

In the following, the relationship between smoking on the one hand and maternal BMI on the other hand and the effect of birth weight will be analyzed. The study aims to show whether there is a general correlation between maternal BMI and nicotine abuse, initially independently of the fetal birth weight. Additionally, the influence of maternal BMI plus nicotine abuse (subdivided into four groups: a) non-smokers; b) 1–7 cigarettes/day; c) 8–14 cigarettes/ day; and d) ≥ 15 cigarettes/ day) on fetal birth weight will be investigated.

## Methods

The current data were achieved from the German Perinatal Survey. This Perinatal Survey was established 1970 in Munich and is an important tool for data-supported quality assurance and is conducted throughout Germany [18, 19]. Voluntary participation, anonymity of the patient, her child and the participating clinic, as well as confidentiality are important keywords in data collection [18]. Thus, it is possible to identify quality features and indicators, location determination and to identify potential for improvement [18]. For example, the following patient data are collected and coded by the responsible doctor and midwife: Delivery mode (spontaneous delivery with respective birth injury, vacuum extraction, forceps, primary/ secondary/ emergency Caesarean section), maternal diseases during pregnancy, such as pre-eclampsia or HELLP-syndrome; concomitant diseases, BMI, nicotine abuse.

The database of Schleswig-Holstein comprised 110.047 singleton pregnancies which were registered between 2010 and 2017. The evaluation took place in August 2018. Informed consent was given by all patients in written form. The BMI was taken from the maternity log, where the height and weight was measured by the resident gynecologist. Information concerning the smoking status were self-reported by the patient. Based on their smoking status during pregnancy, patients were divided into smokers and non-smokers and assigned to one of the following four groups according to their daily smoking behavior: a) non-smokers; b) 1–7 cigarettes/day; c) 8–14 cigarettes/ day; and d) ≥ 15 cigarettes/ day [19].

Furthermore, we classified women by their BMI into underweight (BMI ≤ 18.5 kg/m^2^), normal weight (BMI 18.5–24.99 kg/m^2^), overweight (BMI 25.0–29.99 kg/m^2^) and obese (BMI ≥ 30 kg/m^2^).

Depending on maternal cigarette consumption und BMI, we determined the neonatal outcome according to the infants’ somatic development based on their birth weight.

The data are shown as percentages or absolute numbers as indicated. The t-test was used for comparisons between non-smokers and the respective smoking group, and their relationship with maternal BMI. The level of significance was set to 5%. Statistical analysis was performed at the data center of the University of Rostock using the SPSS computer program, version 22.0 [19].

## Results

**Fig. 1:**
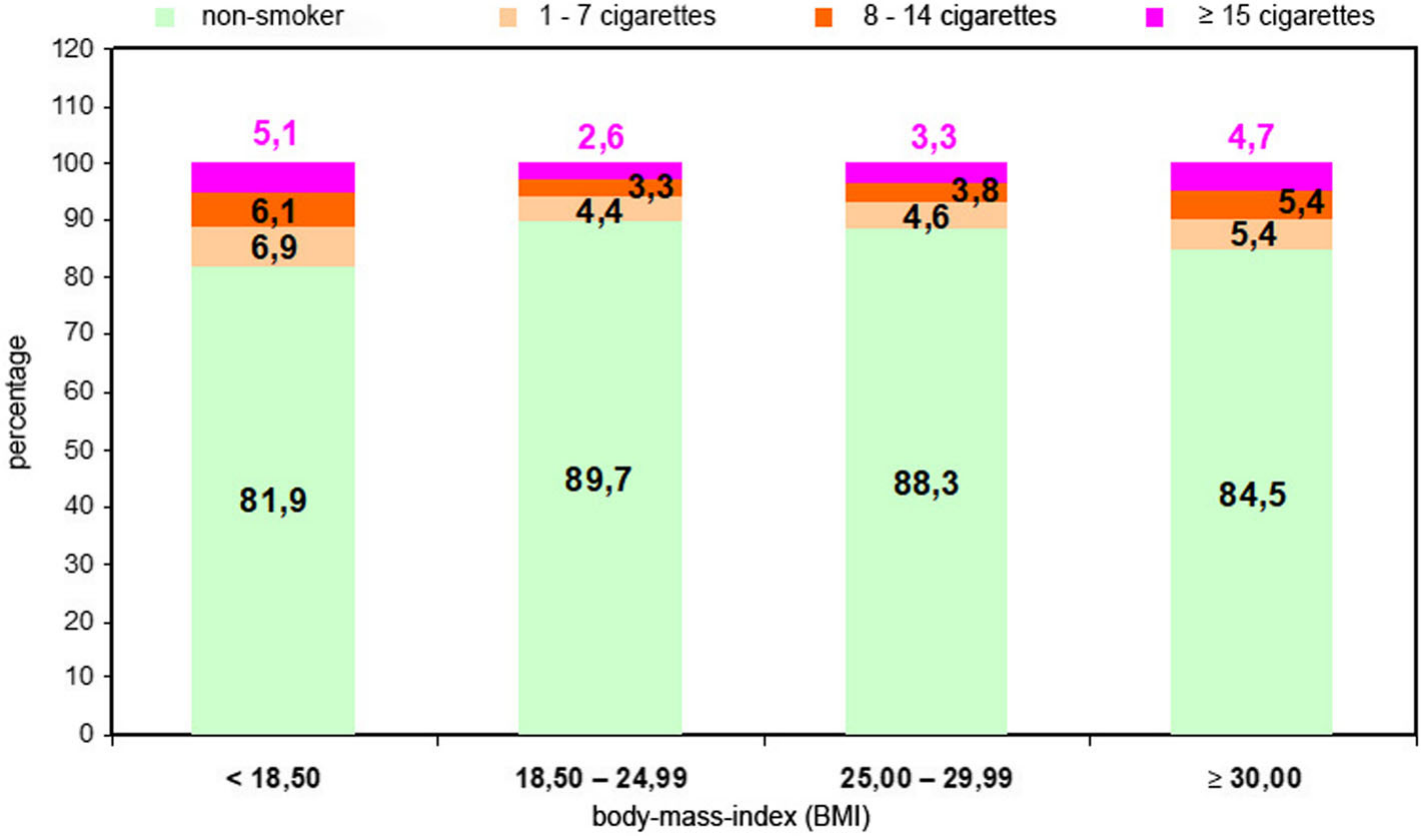
Relationship between maternal BMI and the number of cigarettes smoked daily (in %); n (total) = 110.047

The investigation consisted of 110.047 pregnant women. Of these, 97.092 (88.2%) were non-smokers and 12.955 (11.8%) smokers. The prevalence of maternal smoking and their BMI were interrelated. 10.3% of normal-weight women smoked during pregnancy, but both high and low BMI were associated with a higher prevalence of smoking. The proportion of smokers was highest (18.1%) among underweight women (BMI ≤ 18.5 kg/m^2^). In addition, the proportion of women who smoked the maximum number of cigarettes were also registered in this group (5.1% of women smoked ≥15 cigarettes and 6.1% smoked 8–14 cigarettes; of normal-weight women 2.6% smoked ≥15 cigarettes and 3.3% smoked 8–14 cigarettes). The smoking behavior of overweight women (BMI 25.0–29.99 kg/m^2^) was similar to that of the normal-weight group, but nevertheless a little higher (11.7% vs. 10.3%). A large percentage of smokers (15.5%) was noted in the obese group (BMI ≥ 30 kg/m^2^). Figure 1 shows the maternal BMI and the numbers of cigarettes smoked daily divided into non-smokers, 1–7 cigarettes/day, 8–14 cigarettes/day and 15 or more cigarettes/day.

**Fig. 2:**
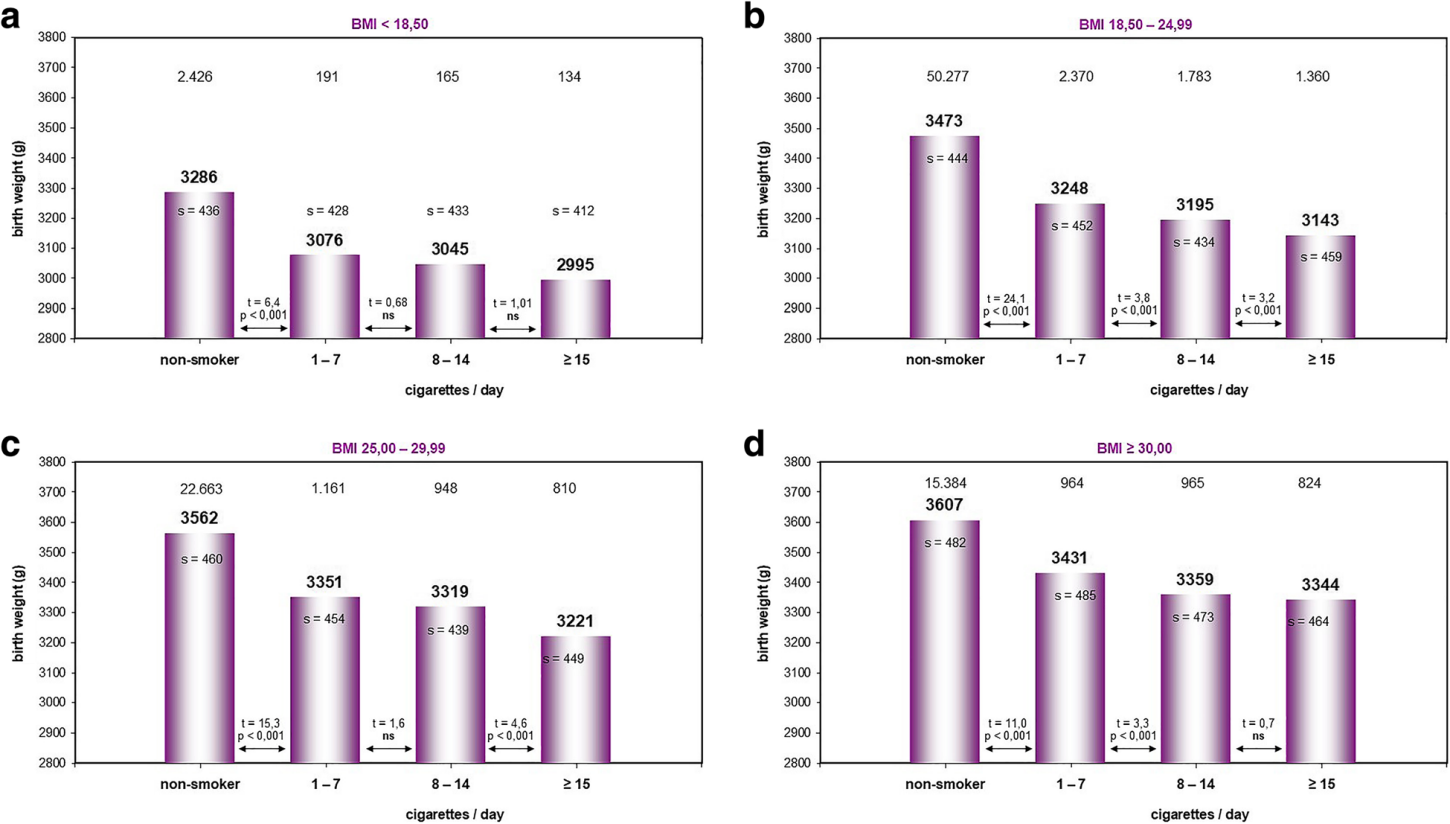
**a-d** Impact of maternal smoking and BMI on mean birth weight ≥ 37 + 0 gestational age; n (total) = 102.425; s = standard deviation, ns = not significant, t = t distribution

Smoking and BMI interact with regard to perinatal outcome. In Fig. 2a-d we investigated the infants’ birth weight in relation to the mothers’ daily consumption of cigarettes. In this analysis, we included all women and newborn delivered ≥37 + 0 gestational age in order to avoid a distortion of statistics. This new group consisted of 102.425 pregnant women. Of these, 90.750 (88.6%) were non-smokers and 11.675 (11.4%) smokers.

In both smokers and non-smokers, we noted a dependence of mean birth weight on maternal BMI. Mean birth weight increased with increasing maternal BMI, and was reduced by smoking for every BMI category. The differences between smokers and non-smokers were always highly significant (*p* < 0.001). Mean birth weight varied between 2995 g in underweight frequent smokers and 3607 g in obese non-smokers.

Figure 2a summarizes the data of underweight women (BMI ≤ 18.5 kg/m^2^), *n* = 2.916. Birth weight was highest among non-smokers (3286 g), but nevertheless lowest compared to all other BMI categories and non-smoker groups. The infants’ birth weight was significantly higher than that registered in women who smoked 1–7 cigarettes/day. Interestingly, although birth weight tended to decrease with the number of cigarettes smoked, the data did not achieve statistical significance.

Figure 2b summarizes the data of normal weight women (BMI 18.5–24.99 kg/m^2^), *n* = 55.790. Again, birth weight was highest among non-smokers (3473 g). In all groups, birth weight decreased significantly with the number of cigarettes smoked per day (*p* < 0.001).

Figure 2c shows the data of overweight women (BMI 25.0–29.99 kg/m^2^), *n* = 25.582. Birth weight was highest among non-smokers (3562 g). The differences in birth weight between non-smokers and smokers (1–7 cigarettes/day), as well as between smokers (8–14 cigarettes/day) and frequent smokers (> 15 cigarettes/day) was significant (*p* < 0.001).

Figure 2d refers to obese women (BMI ≥ 30 kg/m^2^), *n* = 18.137. Birth weight was highest among non-smokers (3607 g) and also significantly higher than that in women smoking 1–7 cigarettes/day (*p* < 0.001). Comparing smokers of 1–7 cigarettes/day with those who smoked 8–14 cigarettes/day, it was found that the infants’ birth weight decreased as well significantly when the mothers were more frequent smokers (p < 0.001).

## Discussion

In our study nearly 12% of women smoked during pregnancy. The percentage of smokers among underweight women was about twice as high as that among women of normal weight (18.1% vs. 10.3%). In addition, the number of cigarettes smoked daily was highest among underweight (5.1%) and obese women (4.7%) compared to women of normal weight (2.6%).

The infants’ mean birth weight increased with rising maternal BMI and was lower among smokers for each BMI category. The differences in mean birth weight between smokers (even among those who smoked just 1–7 cigarettes per day) and non-smokers were always highly significant (*p* < 0.001). The lowest birth weight (mean 2995 g) was registered among underweight women who smoked more than 15 cigarettes per day.

The limitations of the present study are worthy of mention. First, the patients made their own statements about their cigarette consumption; the truth of these statements cannot be established. In addition, we did not determine whether the number of cigarettes smoked was constant throughout the women’s pregnancy or how many cigarettes they smoked in each trimester. Smoking is known to have different effects in different phases of pregnancy [20]. Furthermore, we did not address the issue of passive smoking. Environmental tobacco smoke is liable to influence birth weight. As smokers socialize in places where cigarettes are smoked, passive smoking may have exerted an additional impact on fetal growth [2]. On the other hand, even non-smoking women may be affected by passive smoking if family members smoke in their vicinity. Finally, we did not determine the influence of other lifestyle habits associated with smoking, such as an unhealthy diet or lack of exercise.

Our results are consistent with a number of studies conducted throughout the world. In a Czech study comprising 4530 women, the authors found that the children of mothers who were moderate or heavy smokers during pregnancy had a birth weight that was lower on average by 245 g [21]. In a Brazilian study the authors analyzed 5166 live births in the city of Pelotas, Brazil, during 1993, and found that the birth weight of children whose mothers smoked during pregnancy was 142 g lower than the birth weight of children whose mothers did not smoke [22]. Jaddoe et al. published a study from the Netherlands comprising 7098 pregnant women; the authors analyzed fetal outcomes after active and passive smoking during different phases of pregnancy. The strongest association was found between active maternal smoking in late pregnancy and low birth weight [20].

Another interesting study concerning reduced fetal measurements in ultrasound depending on maternal smoking behavior was published by Abraham et al. [23]. The authors showed that maternal smoking was associated with reduced second trimester head size and femur length and reduced third trimester head size, femur length and estimated fetal weight. Higher maternal cigarette consumption was associated with a lower z score for head size in the second trimesters compared to lower consumption. Fetal measurements were not reduced for those whose mothers quit before or after becoming pregnant compared to mothers who had never smoked [23].

Based on our results, the question arises as to why the proportion of female smokers in the lower and upper BMI groups was higher than that in women with a normal BMI. Smoking is related to a lower socioeconomic status, which is frequently associated with an unhealthy lifestyle. A healthy and balanced diet as well as an active and mobile lifestyle appear to be rare in this group. The greatest impact of nicotine was noted among underweight women who were frequent smokers. This was the group with the lowest birth weight (on average 2995 g). The harmful effect of tobacco smoke is clearly expressed here and is not concealed by a high maternal BMI. The higher the maternal BMI, the higher the fetal birth weight. Among obese women who were heavy smokers, the infants’ birth weight was even higher as that among underweight non-smoking women (3344 g vs. 3286 g). The unborn infants of obese women who smoked heavily were also affected by the toxic substances contained in tobacco, but appeared to be less affected by the higher birth weight resulting from a high maternal BMI. This risk group should be monitored as closely as underweight women who are heavy smokers.

In view of this BMI-dependent distribution of smokers among pregnant women, smoking and BMI outside the normal range may be expected to interact in the causation of adverse perinatal outcomes in a considerable number of women. Interactions of maternal smoking in pregnancy with maternal body weight might also be relevant for later adverse events such as sudden infant death syndrome [24–26]. As mentioned earlier in this report, smoking during pregnancy is associated with many adverse effects. These include restricted intrauterine growth and low birth weight – as mentioned above – but also placenta previa, abruptio placentae, impaired maternal thyroid function, premature rupture of membranes, perinatal mortality, and ectopic pregnancy. The risks of smoking during pregnancy extend beyond pregnancy-related complications. Children born to mothers who smoke during pregnancy are at a higher risk of developing asthma, infantile colic, and childhood obesity [27].

Pineles et al. [28] conducted a systematic review and meta-analysis to characterize the relationship between smoking and miscarriage. The authors showed an increased risk of miscarriage in case of any active smoking (summary relative risk ratio = 1.23, 95% confidence interval (CI): 1.16, 1.30; *n* = 50 studies). Furthermore, the risk of miscarriage increased with the amount of cigarettes smoked (1% increase in relative risk per cigarette smoked per day). A further analysis concerning secondhand smoke showed that exposure during pregnancy increased the risk of miscarriage by 11% (95% CI: 0.95, 1.31; *n* = 17 studies) [28].

Two years later, the same author [29] performed a systematic review and 3-part meta-analysis in order to evaluate the relationship between smoking and perinatal death. Stillbirth as well as neonatal death was correlated with maternal smoking behavior. Any active maternal smoking was associated with increased risks of stillbirth (summary relative risk (sRR) = 1.46, 95% confidence interval (CI): 1.38, 1.54 (*n* = 57 studies)), neonatal death (sRR = 1.22, 95% CI: 1.14, 1.30 (*n* = 28)), and perinatal death (sRR = 1.33, 95% CI: 1.25, 1.41 (*n* = 46)). The higher the number of cigarettes smoked, the greater the risks of stillbirth, neonatal death, and perinatal death. These findings highlight the importance that women should not smoke during pregnancy, and all women of reproductive age should be warned that smoking increases the risks of stillbirth, neonatal death, and perinatal death [29].

## Conclusions

Based on the data mentioned above, we have shown the connection between smoking and maternal BMI and its impact on perinatal outcome. The infants’ mean birth weight increased with rising maternal BMI and was lower among smokers for each BMI category. Smoking during pregnancy was significantly associated with low birth weight. Already a nicotine abuse of 1–7 cigarettes daily has a significant effect on low birth weight compared to non-smokers. Pregnant women should be advised to cease or at least reduce smoking in order to improve the birth weight of the newborn and to minimize child morbidities.

## Data Availability

The datasets analysed during the current study are available from the corresponding author on reasonable request.

## Abbreviations

BMI: body mass index
SGA: small for gestational age
APGAR: Atmung-Puls-Grundtonus-Aussehen-Reflexe
